# Developing a research community within an online healthcare feedback platform

**DOI:** 10.1111/hex.13696

**Published:** 2023-01-20

**Authors:** Beth Fylan, James Munro, Jane K. O'Hara, Binish Khatoon, Rebecca Lawton

**Affiliations:** ^1^ Yorkshire Quality and Safety Research Group Bradford Teaching Hospitals NHS Foundation Trust Bradford UK; ^2^ School of Pharmacy and Medical Sciences University of Bradford Bradford UK; ^3^ Care Opinion Community Interest Company Sheffield UK; ^4^ School of Healthcare University of Leeds Leeds UK; ^5^ School of Health Sciences The University of Manchester Manchester UK; ^6^ School of Psychology University of Leeds Leeds UK

**Keywords:** online community, online feedback, patient involvement, research community, technology platform

## Abstract

**Introduction:**

Care Opinion is an online feedback platform supporting patients to author stories about their care. It is not known whether authors would be willing to be involved in improving care through research. The aims of this study were to explore the views and preferences of Care Opinion authors about joining an online research community and to pilot new research community functionality.

**Methods:**

Five hundred and nine Care Opinion authors were invited to take part in an online survey in June 2019. Survey items included questions about participants' willingness to take part in research and their preferences for supporting processes. Data were analysed descriptively. Authors were invited to consent to join a research community and were asked to participate in three pilot studies.

**Results:**

One hundred and sixty‐three people consented to take part in the survey (32%). Participants indicated they would like to know the time commitment to the project (146, 90%), details about the organization carrying out the research (124, 76%) and safeguarding information (124, 76%). Over half indicated that they did not know how to get involved in healthcare research (87, 53%). Subsequently, 667 authors were invited to join the research community, 183 (27%) accepted, and three studies were matched to their expressed preferences for project attributes or organization type.

**Conclusion:**

Many people who leave online feedback about their experiences of healthcare are also willing to join a research community via that platform. They have strong preferences for supporting University and NHS research. Eligibility and acceptance rates to join pilot research studies varied. Further work is needed to grow the research community, increase its diversity, and create relevant and varied opportunities to support research.

**Patient or Public Contribution:**

Four members of the Safety In Numbers patient and public involvement and engagement (PPIE) group advised about survey development.

## 1 INTRODUCTION

There has been a marked increase internationally in healthcare organizations collecting and responding to feedback from patients about their experiences of health care using different methods.^1^, ^2^, ^3^, ^4^, ^5^ Traditionally, people who have received treatment have had limited routes to provide feedback about the care they have received. For example, face‐to‐face feedback during episodes of care, surveys, letters and cards to care providers, the Patient Advice and Liaison Service and healthcare organization complaints interfaces have been the main routes through which feedback can be provided. New technologies, such as online platforms and social media, now provide formal and informal opportunities for patients to offer open feedback and recount often in‐depth stories. These platforms can provide an understanding of patients' experiences during and after having received care to healthcare teams and to other patients.^6^, ^7^, ^8^ One such online platform is Care Opinion, which supports patients to author stories about care they have experienced and offers the opportunity for staff to respond to those stories, which they do with varying levels of engagement.^9^

The growing popularity of online platforms such as Care Opinion indicates that people are willing to offer feedback about their care in this way. Patients have described being motivated to do so for a range of reasons, including improving the care they and others receive, improving healthcare services, publicly thanking and praising staff or a service, generally to empower patients or to inform other patients.^9^, ^10^ A recent study by Mazanderani et al.^8^ found that those who leave feedback do so as an act of caring about other patients, and caring about the healthcare system and those working in it. Whilst it is established that some people are willing to give feedback to improve services, it is not known whether people who offer this type of feedback are willing to be further involved in improving health care. Given that improving healthcare services is a major motivator for offering feedback, it is possible that those who do so may be willing, if asked, to be further involved in healthcare improvement as participants or advisors to research.

Further, considering the widely reported difficulties in recruiting people into healthcare research studies,^11^, ^12^ exploring new and different routes to recruitment, especially those that may offer the ability to select participants based on their health conditions and their demographics, may offer a solution to many healthcare research projects. Routes to recruitment that do not involve placing an additional burden on stretched healthcare services may be of particular benefit because they would not require the input of clinical teams, for example, in identifying participants, or of healthcare administration staff in the distribution of study recruitment materials. Indeed, during the first two waves of the COVID‐19 pandemic, most non‐Covid related research was discontinued within the NHS, and it was not possible to access patients via healthcare organizations to take part in the research. It is now, more than ever, important that other viable recruitment options are identified.

Involving and engaging users of health care in the design and management of research can also be challenging for researchers, who may default to using established patient and public involvement groups convened by healthcare organizations.^13^ There is an ever‐deeper emphasis on the involvement of patients in healthcare services research to increase its quality and impact. The potential for patients and the public to be involved spans the different stages of research,^14^ from developing priorities and setting the research programme,^15^ during the development of research protocols, assessing the appropriateness of research instruments,^16^ and of course as participants. Further opportunities are available during the data analysis and write‐up stage to ensure that emerging themes are interpreted from lay perspectives.^17^ There is potential for those who are willing to provide feedback about their experiences online to contribute to research, either as participants, advisers or co‐researchers, to improve care. The aims of this study, therefore, were to:

(1) Examine the views and preferences of Care Opinion authors (people who have shared their stories on the Care Opinion platform) about joining an online research community;
(2) Assess whether those who join a research community then agree to take part in research studies, if eligible;
(3) Pilot new research community functionality within the Care Opinion website.

## 2 MATERIALS AND METHODS

We undertook a quantitative study using an online survey method and used the results to inform the development and pilot testing of research community functionality within the Care Opinion platform.

### 2.1 Survey development

The survey was developed by a team of three researchers with backgrounds in applied health and social research (B.F. and B.K.) and psychology (J.K.O'H.), and a Care Opinion team member with a background in clinical medicine, public health and health services research (J.M.). Four members of the Safety In Numbers patient and public involvement and engagement (PPIE) group advised about survey items. Survey items were developed based on a review of the literature about frameworks supporting patient and public involvement in research,^18^ and included items about the participants, for example, their age, ethnicity, gender, and employment status. Survey items also included questions about participants' perception of the importance of healthcare research, their willingness to take part in research, the research‐related activities they would be willing to get involved in, the types of research organizations they would be willing to engage with, whether they would require payment to be involved in research, and their preferences for processes supporting research involvement. Answer options comprised Likert scales, yes/no options and open questions. Two sets of questions, one measuring prosocialness^19^ and another exploring quality in service‐user involvement,^20^ were adapted, although the results of those items are not reported here. Four people piloted the survey and gave feedback on the length of time the survey took to complete and the wording of the survey items. The survey was programmed so that respondents were not forced to answer every question.

### 2.2 Participants and recruitment

Participants were adults who had submitted a story to Care Opinion during May and June 2019 and had consented to further contact from the Care Opinion team. To reduce the risk of causing distress, we excluded authors who described end‐of‐life care or care resulting in serious negative effects on physical or mental health. A recruitment email was sent by Care Opinion inviting people to take part in the study with a link to the questionnaire hosted on the SurveyMonkey site. A link to an information statement about the study and a consent form was embedded at the beginning of the survey. In total, the invitation email was delivered to 509 care opinion authors in July 2019. A reminder email was sent 2 weeks later, and the survey remained open for 1 month.

### 2.3 Data analysis

Descriptive statistics, including counts of responses to questions and associated percentages, were computed using the software programme SPSS version 27. Missing data were not deducted from the total possible responses. Open questions were explored using content analysis. Responses were grouped into categories and counts, and percentages of categories were calculated. In this article, we report responses to a subset of survey items.

### 2.4 Piloting the research platform within Care Opinion

Following the survey analysis, we used the results to inform the design of a ‘proof of concept’ research community on the Care Opinion platform. We added a feature to the platform enabling authors to join the community and express their preferences for communication. A further feature enabled Care Opinion staff to send project invitation emails to research community members, allowing invitees to accept or decline the invitation as they chose.

To create an initial community population, we defined a sample of adults who had posted a story on Care Opinion between February and April 2020, and we emailed those authors with an invitation to join the pilot community. We sent an email a reminder after 1 week. Subsequently, we invited community members to participate in three different types of research study over the following 9 months. The first study sought unselected participants to respond to a survey about medical uncertainty in the emergency department. The second sought interviewees for a study of experiences of patients and family members involved in a serious incident in an NHS hospital or mental health service in England. For both of these studies, we sent automated email invitations via Care Opinion to community members whose consent preferences matched the study (e.g., research organization, type (survey, interview), location (online, phone, home visit). For the second study, eligibility was set out in the invitation. The third study required interviewees who were over 75 and had experienced a recent hospital stay, or they were a family member or carer of such a person. Given the very narrow requirements of this study, and after an initial automated email invitation to research community members detailing eligibility produced disappointing results, we undertook a wider search of stories posted in the past year (i.e., beyond our research community cohort) which was then manually filtered to identify potentially suitable authors for an email invitation.

## 3 RESULTS

A total of 163 people (32%) consented to take part in the survey. Of these, 110 were female (67.4%) and 52 were male (31.9%). This reflects the population of Care Opinion authors, which has a higher proportion of women compared to men (64% vs 36%). Most respondents were white (151; 92.6%), and over a third were retired (57, 34.9%). Respondents were of different ages, with 55–64 being the largest age group. A quarter of respondents were under 45 years old. The majority of respondents were over 55 years old (91, 55%). This compares well to the population of Care Opinion authors, where the median age is 55. The demographics of survey respondents are shown in Table 1. Most participants had used the Care Opinion site to leave a story about their own care (115; 70.6%), 37 had left a story about a relative's care (22.7%), and the remainder had posted a story about the care of a partner (8, 4.9%) or friend (1, 0.6%).

**Table 1:** Demographics of survey respondents (base *n* = 163)

	Number (%)
Gender	
Female	110 (67.4)
Male	52 (31.9)
Missing	1
Age range	
18–24	4 (2.5)
25–34	18 (11.0)
35–44	20 (12.3)
45–54	30 (18.4)
55–64	50 (30.7)
65+	40 (24.5)
Missing	1
Ethnicity	
White	151 (92.6)
Asian or British Asian	5 (3.1)
Mixed race	2 (1.2)
Black or Black British	1 (0.6)
Other	3 (1.8)
Missing	1
Employment status	
Retired	57 (34.9)
Full‐time employed	52 (31.9)
Part‐time employed	24 (14.7)
Not currently employed	10 (6.1)
Homemaker	3 (1.8)
Education	2 (1.2)
Other (e.g., carer, volunteer, self‐employed and disabled)	15 (9.2)

### 3.1 Views about healthcare research

The vast majority of the 161 people who answered the question about whether healthcare research was important, reported that it was very (149, 91.4%) and fairly (12, 7.4%) important. Table 2 shows responses to three further questions about taking part in healthcare research. The majority either agreed or strongly agreed that taking part in healthcare research improves health care (156, 95.7%), and agreed or strongly agreed that patient and public involvement in healthcare research is important (160, 98.2%). Over half of respondents either agreed or strongly agreed that they did not know how to get involved in healthcare research (87, 53.4%).

**Table 2:** Responses to questions about taking part in healthcare research (base *n* = 163)

	Strongly agree	Agree	Neither agree nor disagree	Disagree	Strongly disagree	Missing
Taking part in healthcare research improves healthcare	111 (68.1%)	45 (27.6%)	5 (3.1%)	2 (1.2%)	0	0
Patient and public involvement in healthcare research is important	130 (79.8%)	30 (18.4%)	1 (0.6%)	0	0	2
I don't know how to get involved in healthcare research	22 (13.5%)	65 (39.9%)	39 (23.9%)	28 (17%)	7 (4.3%)	2

### 3.2 Taking part in a research project

Answers to questions about how authors would respond to an invitation to take part in a research project are in Table 3. Most respondents reported that they would probably or definitely not immediately opt out (132, 80.9%). Most respondents would probably or definitely agree to help University research (122, 87.1%) and even more would probably or definitely agree to help NHS research (151, 92.6%), whilst less than half would probably or definitely agree to help research by private companies (71, 43.5%). The majority would be happy to consider invitations about any kind of research (103, 63.2%), but NHS research received the most support and private companies the least.

**Table 3:** How Care Opinion authors would respond to an invitation to take part in a research project (base *n* = 163)

	Definitely not	Probably not	Not sure	Probably	Definitely	Missing
I would opt out immediately	68 (41.7%)	64 (39.3%)	22 (13.5%)	4 (2.5%)	1 (0.6%)	4
I would only want to receive invitations about research which related to the story I posted	35 (21.5%)	42 (25.8%)	24 (14.7%)	49 (30.1%)	12 (7.4%)	1
I would agree to help university research	2 (1.2%)	2 (1.2%)	16 (9.8%)	88 (54%)	54 (33.1%)	1
I would agree to help NHS research	4 (2.5%)	1 (0.6%)	6 (3.7%)	77 (47.2%)	74 (45.4%)	1
I would agree to help research by private companies	10 (6.1%)	16 (9.8%)	65 (39.9%)	55 (33.7%)	16 (9.8%)	1
I would be happy to consider invitations about any kind of research	4 (2.5%)	17 (10.4%)	39 (23.9%)	68 (41.7%)	35 (21.5%)	0
I would only help research if I could do it online	21 (12.9%)	29 (17.8%)	59 (36.2%)	36 (22.1%)	17 (10.4%)	1

### 3.3 Information required about the research

The information participants would like before taking part in the research is in Table 4. The most frequently selected type of information they would like was the time commitment to the project (90% of respondents selected this option), followed by details of the organization carrying out the research, safeguarding information, travel commitments and timelines.

**Table 4:** Information respondents would like about the research before taking part (base *n* = 163)

Information respondents would like before deciding whether to be involved	Number (%)
Your time commitment to the project	146 (89.6)
Details about the organization carrying out the research	124 (76.1)
Safeguarding information for you if you took part	124 (76.1)
Travel commitments/locations	119 (73)
Timelines for the project	111 (68.1)
Key people involved in research	74 (45.4)
Details about payments for your contribution to the project	51 (31.3)

*Note*: Participants could select multiple options to this question.

### 3.4 Respondents' views about using the Care Opinion platform for research

We asked a series of questions about preferences for taking part in research via the Care Opinion platform to guide the design of the research platform. Respondents' answers are in Table 5. The majority of respondents agreed or strongly agreed that people should be able to opt‐in or out of being asked to help with research (156, 95.7%), that people should be able to limit how often they are asked to help with research (130, 79.7%), that people should be able to see what kind of organization is asking for their help (158, 96.8%), and that people should be able to read information about the research before they decide whether to help (153, 93.9%).

**Table 5:** Respondents' views and preferences for taking part in healthcare research (base *n* = 163)

	Strongly agree	Agree	Neither agree nor disagree	Disagree	Strongly disagree	Missing
People who share a story on Care Opinion may have experiences which would help research studies	112 (68.7%)	44 (27%)	6 (3.7%)	0	0	1
People who share a story on Care Opinion would not want to help research	5 (3.1%)	1 (0.6%)	38 (23.3%)	76 (46.6%)	41 (25.2%)	2
I would like to see people who share stories on Care Opinion also helping research	80 (49.1%)	64 (39.3%)	17 (10.4%)	0	0	2
I would like to be able to see if a particular story author has also helped a research study	41 (25.2%	57 (35%)	48 (29.4%)	14 (8.6%)	1 (0.6%)	2
If I helped a research study, I would like people to be able to see that on Care Opinion	45 (27.6%)	51 (31.3%)	47 (28.8%	16 (9.8%)	2 (1.2%	2
I don't think Care Opinion should be inviting people to help research	4 (2.5%)	3 (1.8%)	17 (10.4%)	73 (44.8%)	64 (39.3%)	2
I am more likely than other people to help research because I am an author on Care Opinion	18 (11%)	53 (32.5%)	58 (35.6%)	22 (13.5%)	10 (6.1%)	2
People should be able to opt in or out of being asked to help	94 (57.7%)	62 (38%)	3 (1.8%)	2 (1.2%)	1 (1.2%)	1
People should be able to limit how often they are asked to help	60 (36.8%)	70 (42.9%)	19 (11.7%)	9 (535%)	3 (1.8%)	2
People should be able to see what kind of organization is asking for their help	110 (67.4%)	48 (29.4%)	3 (1.8%)	0	0	2
People should be able to read information about the research before they decide to help or not	110 (67.5%)	43 (26.4%)	6 (3.7%)	3 (1.8%)	0	1

### 3.5 Types of research people would take part in

More than 80% of respondents answered the open question about the research they would support, and some offered multiple types of research in their answers. Responses are in Table 6. Many respondents (59, 36.2%) gave answers indicating that they would support research relevant to their own condition or story, or a specific health condition such as arthritis, mental health conditions, types of cancer and chronic pain or ageing. Respondents also indicated they would support research relating to services, such as rehabilitation, improving services in care settings such as hospitals, and management of the NHS and social care (26, 16%). Others indicated they would like to help with research that supported the patient experience, patient satisfaction, perceptions of treatment and what matters to patients and families (13, 8%). A small number would support research focussing on a particular group, such as children, older people and ethnic minorities (5), whilst four people indicated they would support a particular research type, such as qualitative, surveys or research to improve methods. The remainder of responses (3) related to health promotion, and stem cell research.

**Table 6:** Types of research participants would and would not help with and concerns about being asked to take part (base *n* = 163)

Research participants would help with	Number (%)	Research participants would not help with	Number (%)	Concerns about being asked to take part in research	Number (%)
Research relevant to own condition or story	59 (36.2)	None	84 (51.5)	None	70 (42.9)
None/unsure/any	38 (23.3)	Profit‐making/private sector	8 (4.9)	Resources	18 (11.0)
Research related to services	26 (16.0)	Clinical, invasive or drug research	7 (4.3)	Privacy	15 (9.2)
Research supporting patient experience	13 (8.0)	Research that will not change anything	3 (1.8)	Opting in and out	6 (3.7)
Research focussing on specific group	5 (3.1)	Animal research	3 (1.8)	Legitimacy	5 (3.1)
Research using a specific method	3 (1.8)	Learning disability research	2 (1.2)	Ethics	4 (2.5)
Other	3 (1.8)	Children/childhood research	2 (1.2)	Lack of impact	2 (1.2)
		Time‐consuming research	2 (1.2)	Distract from Care Opinion's main objectives	1 (0.6)
		Addiction research	1 (0.6)		

The majority responded to the open question about the research they would support (69%), and the answers covered more than one type of research. Most indicated there was no research they would not help with (84, 51.5%). Eight people responded that they did not want to support profit‐making or private‐sector research (4.9%), and seven people did not want to support clinical, invasive or drug research (4.3%). Other responses included research that would not bring about change or that they could not contribute to. Three people did not want to support research involving animals, two people did not want to support learning disability research and two people did not want to support research with children or about childhood. Two people indicated that they would not help with research that took too much time. Finally, one person did not want to help with addiction research.

Nearly three‐quarters (71.2%) of participants answered the open question about whether they had concerns about being asked to take part in research, and their answers covered multiple concerns. Many had no worries (42.9%). Eighteen people had concerns relating to resources, for example, travel, the time it would take, or being bombarded with requests (11%). Fifteen people had worries about privacy, for example, being identified or judged, and data security (9.2%). Seven people had concerns about being able to opt‐in or out (3.7%). Other concerns related to the legitimacy of organizations carrying out the research, ethical issues, such as other people's motivations for taking part in research and the emotional burden on participants, that the research would lack impact or that research would impact Care Opinion's main objectives.

### 3.6 RESULTS OF THE PILOT TEST OF THE RESEARCH COMMUNITY

Of the 667 authors invited to join the research community, 183 (27.4%) accepted, and 5 (0.7%) declined. On joining, and based on survey responses, authors were able to set their preferences for participation (research organization type, acceptable communication channels). We invited members of this community to participate in three research projects. For the first two research studies, we sent automated email invitations via Care Opinion to community members whose expressed preferences matched the project attributes. For the first research project, a survey of views on medical uncertainty, we invited 128 research community members whose preferences matched the project. Of these, 67 authors (52.3%) accepted the invitation, and 49 (38.2%) went on to complete the survey. For the second, a more specific project, we invited 139 members willing to receive invitations from a university, of whom 11 authors (7.9%) were eligible and accepted the invitation whilst 30 (21.5%) declined. This project required participants to have experience a ‘serious incident’ in their care.

For the third project, seeking people over 75 with a recent hospital episode, or their carers, 23 research community members were invited, of whom 2 (8.7%) accepted an invitation. To extend the sample, we undertook a wider search of Care Opinion stories (not restricted to research community authors) posted in the past year, which included the words (‘elderly’ or ‘old*’) and (‘ward’ or ‘discharge’). This resulted in 441 candidate stories, of which 52 were tagged as relevant by the researcher. The authors of these stories were invited to participate by email, resulting in seven further authors (13.5%) agreeing to interview.

## 4 DISCUSSION

This project examined the views and preferences of Care Opinion authors about joining an online research community and pilot‐tested a research community within the Care Opinion platform. It found that survey respondents perceived healthcare research to be important, that patient and public involvement in research is important, that the majority would be willing to help with any type of research and thought that people who shared a story on Care Opinion had experiences that would help research studies. People reported that they should be able to see the organization asking for their involvement and that they should be able to read information about research before deciding. Respondents were more positive about helping NHS and university research than other types of research, and respondents expressed clear preferences for being able to opt‐in and out and limiting the number of invitations received. They wanted to know the expected time commitment to a project in advance and about safeguarding information. Based on the positive responses of Care Opinion authors about being involved in research, the results of the survey were used to develop a pilot research community platform. Over a quarter of those invited joined the community and over a third of those invited to take part in the first study did so. Finding participants for studies with more specific inclusion criteria was more challenging.

### 4.1 Motivation and barriers to taking part in research

This research found that whilst some survey respondents would support research relevant to their own condition, others were happy to support any research, and some who went on to join the research community did indeed respond to a request to respond to a general survey during the pilot. Previous research has found that people's motivation to take part in research is primarily altruistic.^21^ However, Bradley et al.^22^ found that patients joining research teams were motivated by their own individual needs and wanted to get involved in research that was relevant to their own health condition. Taking part in research was viewed by their participants as contributing towards the public good and the trustworthiness of researchers was judged based on their organization and profession. Further, Dixon‐Woods and Tarrant (2009)^23^ discussed how people may be reluctant to join research if they are sceptical of the intentions of the researchers, especially when there is a cost to them in the form of, for example, providing personal data or undergoing tests. Respondents in this study indicated that they were more positive about supporting research conducted by the NHS or a university than research by private organizations, which emphasizes the importance of trust to Care Opinion authors. The involvement of  patIent organizations has been found to have a positive impact on participant recruitment,^24^ so clearly, the perceived legitimacy of research is influenced by the organizations conducting and supporting it.^25^ Indeed, a recent review indicated that being part of a trusted research team is crucial to successful patient involvement in research.^26^ It is possible, then, that the overall legitimacy of such a research community may be damaged by allowing access to organizations outside the UK NHS and higher education. Further consultation around this area will be needed before wider access is considered.

Participants in the survey were overwhelmingly positive about being involved in research, yet when authors were invited to join a community, only 27% did so. The dissonance between expressed intentions to get involved in research and people's actions when invited to do so align with behavioural models, such as the theory of planned behaviour,^27^ which proposes an ‘intention‐behaviour gap’ between the intention, formed through attitudes, norms and perceived behavioural control, and the desired action. Achieving a conversion rate of fewer than one‐third of invited authors indicates that barriers to joining for those who are willing need to be fully understood and addressed. Furthermore, as few authors matched our third pilot project—aimed at people over 75 with a recent hospital episode—the community would need to expand to yield sufficient participant numbers for projects with detailed and specific eligibility criteria. More consideration is also needed about facilitating certain groups, such as older populations, to be involved. Iteratively developing recruitment methods may support increased participation,^28^ and involving members of the research community in developing these methods will be important. A systematic review has demonstrated patient and public involvement to improve participant recruitment to studies, particularly if the people involved have experience relevant to the subject of the research, such as the experience of a particular health condition.^29^

Nevertheless, less than 1% of invited authors declined to join the research community, indicating that there was limited direct opposition to doing so, rather than that there might be a lack of internal or external motivation to join on receipt of the invitation. Similarly, how authors are matched to research opportunities requires further consideration. The completion rate for the first pilot study, a general survey, was 38%, however, for the second study, for which the eligibility criteria were specific and related to experiencing a serious incident in care, only 8% accepted the invitation to take part once those criteria had been communicated, and many more (22%) declined. Further information is needed to understand the reason for those declines. It is, of course, possible that more challenging topic areas, particularly for people who have had negative experiences of care or, indeed, have been harmed by their care, may lead to reluctance to take part. In addition, it was not possible to know the health status of people invited to join the community or to take part in pilot studies, so fluctuating health may also have been a barrier.

More than three‐quarters of survey respondents indicated that information about safeguarding would be required before they agreed to take part in a study, and some also had worries about privacy, for example, being identified or judged. Interestingly, there were a large number of definitive declines to the second pilot study about serious incidents, where potential participants may have had fears about the consequences of being identified. Other studies have found that a range of fears, including confidentiality, act as barriers to taking part in research, albeit in clinical trials.^30^ Some survey respondents in this study also had practical concerns about being involved in research or would want information about practicalities involved in taking part, such as time commitments and travel arrangements. Such practicalities can be a barrier to participation, even for those who are motivated to take part.^31^ Reducing the burden of taking part should therefore be a priority for researchers.

### 4.2 Extending the role of online feedback platforms

This work has demonstrated that care feedback platforms are a potential route to engage people in research and can be used to augment other methods of data collection and analysis. Internationally, online feedback routes are increasingly available,^32^ although only to those who have the resources to be able to access them. Dudhwala et al.^33^ drew a distinction between feedback routes that are approved by healthcare organizations and used by them to solicit feedback, and those that are unofficial and not actively used to ask for or review patient feedback. Care Opinion primarily falls into the first of these categories and it is possible that its more established role as a broker of patient feedback heightens trust. As the number of people leaving online feedback grows, the opportunities to involve contributors are also increasing. Improving care is a strong motivation for sharing online feedback, so it is not surprising that our study found that people who posted online feedback also thought that healthcare research is important and that it can improve care. Other research has shown that the motivation for leaving online feedback is to care for or empower other patients and to care for the NHS.^7^, ^8^, ^10^ Care Opinion authors in this study seemed similarly motivated. However, over half of survey respondents did not know how to get involved in healthcare research, indicating that platforms such as Care Opinion could play a valuable role in linking those willing to give their opinions and recount their experiences of healthcare with organizations needing access to participants to deliver their research. There are obvious caveats for researchers to consider, including the need to appraise potential bias introduced by recruiting solely from those who are already willing and have the confidence to author stories online about their care. Further, trust in research conducted via an online feedback platform needs to be explored, especially amongst healthcare staff who can perceive online feedback platforms poorly, for example, as overly negative and not useful in improving care.^34^

### 4.3 Recommendations for future research

This research was conducted within the National Institute for Health and Care Research Yorkshire and Humber Patient Safety Translational Research Centre, and more feasibility work needs to be conducted before broadening the reach of the platform to other researchers. To build on this research, further work should take place to increase the number of authors who accept an invitation to join the community and to increase motivation to take part in research by providing interesting and varied research involvement and participation opportunities. Care needs to be taken to increase the diversity of the research community beyond those who responded to the survey, who were majority white and over 55. Finally, to fully develop the research community, more work needs to be done, based on the results of the survey reported here, to develop functions to allow Care Opinion authors to set additional and more specific preferences about the type of research they wish to be involved in, which will support better‐targeted research invitations.

### 4.4 Limitations

Those who took part in the survey were mostly female, white, retired or employed, and 55 years or older, which is not representative of the population as a whole, although the majority of healthcare users are older and female, and participants were more representative of the population of Care Opinion authors. Demographics of non‐responders to the survey are not available, which is also a limitation. In addition, whilst the research community was piloted within Care Opinion, a formal feasibility study was not conducted so the experiences of those who took part in the pilot testing of the research functionality and subsequent research projects were not explored. The three pilot research invitations were limited in that they invited Care Opinion authors to be participants in research, rather than to be involved in the co‐production of research or other involvement activities. This work was conducted with people who had experienced care in the UK, which is predominantly provided by the National Health Service (NHS). Levels of satisfaction with the NHS at the time of data collection were relatively high (60% very or quite satisfied),^35^ which might have influenced people's willingness to be involved in NHS‐based research. Satisfaction with the NHS has since fallen.

## 5 CONCLUSION

Many people who leave online feedback about their experiences of health care on the Care Opinion platform are also willing to join a research community via that platform and take part in the research. They have strong preferences for supporting NHS and university research. Acceptance rates to join pilot research studies varied, and further work is needed to grow the research community, increase its diversity, create relevant and varied opportunities and reduce barriers to taking part.

## AUTHOR CONTRIBUTIONS

Beth Fylan drafted the manuscript, analysed the data and contributed to research and online community design. James Munro drafted the manuscript, contributed to the research design, led online community development and facilitated the research pilot projects. Jane K. O'Hara led the research and contributed to online community development. Binish Khatoon drafted the research protocol, drafted the survey and commented on the manuscript. Rebecca Lawton advised on online community development, oversaw the pilot research projects and commented on the manuscript.

## CONFLICT OF INTEREST

James Munro is Chief Executive Officer of Care Opinion CIC.

### 1 ETHICS STATEMENT

Ethical approval was granted by the Biomedical, Natural, Physical and Health Sciences Research Ethics Panel at the University of Bradford on 10/04/2019—reference E721.

## Data Availability

The survey data reported in this article are not publicly available to meet the terms of ethical approval for the study and to protect participant privacy.
